# Bariatric Surgery and Type 1 Diabetes: Unanswered Questions

**DOI:** 10.3389/fendo.2020.525909

**Published:** 2020-09-18

**Authors:** Emmanouil Korakas, Aikaterini Kountouri, Athanasios Raptis, Alexander Kokkinos, Vaia Lambadiari

**Affiliations:** ^1^Second Department of Internal Medicine, Medical School, Attikon University Hospital, National and Kapodistrian University of Athens, Athens, Greece; ^2^First Department of Propaedeutic Medicine, Medical School, Laiko General Hospital, National and Kapodistrian University of Athens, Athens, Greece

**Keywords:** bariatric surgery, type 1 diabetes mellitus, obesity, weight loss, glycosylated hemoglobin, GLP-1, insulin sensitivity

## Abstract

In recent decades there has been an alarming increase in the prevalence of obesity in patients with type 1 diabetes leading to the development of insulin resistance and cardiometabolic complications, with mechanisms poorly clarified. While bariatric surgery has long been considered an effective treatment option for patients with type 2 diabetes, the evidence regarding its benefits on weight loss and the prevention of complications in T1DM patients is scarce, with controversial outcomes. Bariatric surgery has been associated with a significant reduction in daily insulin requirement, along with a considerable reduction in body mass index, results which were sustained in the long term. Furthermore, studies suggest that bariatric surgery in type 1 diabetes results in the improvement of comorbidities related to obesity including hypertension and dyslipidemia. However, regarding glycemic control, the reduction of mean glycosylated hemoglobin was modest or statistically insignificant in most studies. The reasons for these results are yet to be elucidated; possible explanations include preservation of beta cell mass and increased residual function post-surgery, improvement in insulin action, altered GLP-1 function, timing of surgery, and association with residual islet cell mass. A number of concerns regarding safety issues have arisen due to the reporting of peri-operative and post-operative adverse events. The most significant complications are metabolic and include diabetic ketoacidosis, severe hypoglycemia and glucose fluctuations. Further prospective clinical studies are required to provide evidence for the effect of bariatric surgery on T1DM patients. The results may offer a better knowledge for the selection of people living with diabetes who will benefit more from a metabolic surgery.

## Introduction

In recent decades, the prevalence of obesity and metabolic syndrome among patients with type 1 diabetes mellitus (T1DM) has alarmingly increased. In a study by Liu et al., the percentage of obesity among patients with diabetes aged 3–19 years old in the U.S. was 12.6%, with another 22.1% found to be overweight (1). The prevalence of obesity was even higher in a study by Price et al. (2), reaching up to 17.2%, with 38.3% of the 501 adults studied found to be overweight. Many other studies have shown similar results regardless of the age of the patients studied (3–6); in general, ~50% of patients with T1DM are overweight or obese, with 8–40% meeting the diagnostic criteria for metabolic syndrome (7). The reasons for this kind of epidemic have not been clarified; however, it seems that intensive insulin regimens to achieve optimal glycemic targets along with the frequent use of simple sugars by the patients to overcome hypoglycemic episodes are the main factors. Obesity is a major threat for patients with T1DM as it triggers a vicious cycle where weight gain leads to insulin resistance which, in turn, leads to increased insulin requirements to achieve glycemic control, resulting in further weight gain. Furthermore, obesity accelerates the occurrence of T1DM at a younger age in patients with a genetic predisposition to the disease (8, 9), while it is related to an increased rate of micro- and macrovascular complications (2, 10).

Bariatric surgery has long been considered an effective treatment for patients with obesity and type 2 diabetes mellitus (T2DM). In 1995, Pories et al. (11) were the first to demonstrate that 82.9% of patients with T2DM and 98.7% of patients with impaired glucose tolerance (IGT) achieved euglycemia for a follow-up period of 14 years after bariatric surgery without antidiabetic medication, and similar results were shown in a study by Buchwald et al. (12), where improvement on glycemic control or total remission of diabetes was observed in 86.6% of the patients studied. As the beneficial metabolic effects on these patients were attributed mainly to weight loss and the concomitant enhancement of insulin sensitivity, the application of bariatric surgery in patients with obesity and T1DM was discouraged, since insulin deficiency rather than insulin resistance was regarded as the main pathophysiologic mechanism of the disease. Nevertheless, the fact that improvements in glycemic control in T2DM patients take place in the course of the first few days after the surgery, before substantial weight loss is achieved, implies that other, weight-loss-independent mechanisms are also responsible for the metabolic amelioration (13). Data on the effects of bariatric surgery in T1DM is scarce, based mainly on case series with relatively few patients and several case reports, and results are conflicting. In this review, our primary end points are to present the existing data about the results of bariatric surgery on T1DM patients concerning weight loss, reduction in insulin requirements, changes in glycosylated hemoglobin (HbA1c) and metabolic comorbidities, along with the main complications and risks, especially diabetic ketoacidosis (DKA) and hypoglycemia. We then discuss the physiological pathways through which bariatric surgery exerts its beneficial effects and special concerns regarding T1DM patients.

## Bariatric Surgery Outcomes in Type 1 Diabetes Mellitus

Bariatric surgery procedures used to be classified as either restrictive or malabsorptive, depending on their mechanism of action; however, this terminology has been abandoned, as it has been shown that each procedure exerts its effects through multiple mechanisms. The main procedures include (a) adjustable gastric banding (AGB) where an adjustable gastric band is placed around the upper part of the stomach to reduce the stomach size, (b) vertical sleeve gastrectomy (VSG), where a tubular stomach is created by partial gastrectomy of the greater curvature side, (c) Roux-en-Y gastric bypass (RYGB), where a small gastric pouch is created from the proximal portion of stomach and is connected to the distal end of the small intestine by gastro-jejunal anastomosis, while entero-enteral anastomosis 100–150 cm distal to the gastro-jejunostomy site restores bowel continuity between the bilio-pancreatic limb and the alimentary limb, and (d) biliopancreatic diversion (BPD) with or without duodenal switch, where gastrectomy is done to reduce the stomach capacity, the stomach is connected to the distal small intestine and the excluded bilio-pancreatic limb is connected to the alimentary limb distally. The most commonly applied procedures are RYGB and VSG (14). However, all these techniques have been mainly studied in T2DM; studies that refer to T1DM are mostly retrospective, with a limited number of patients with heterogeneous characteristics. The most beneficial results are observed in terms of weight loss and daily insulin requirements, while data about improvements in HbA1c demonstrate modest and contradictory outcomes. The main results are summarized in Table 1.

**Table 1:** Effects of bariatric surgery on weight loss, daily insulin requirements and glycosylated hemoglobin in patients with obesity and type 1 diabetes mellitus.

**References**	**Patients (n)**	**Mean duration of T1DM (years)**	**Mean duration of follow-up**	**Procedures (*n*)**	**Mean BMI loss (kg/m^**2**^)**	**Alteration in insulin requirements IU/day (u/kg/d)**	**Alterations in HbA1C**
Czupryniak et al. (15)	3	11.6	75.6 months	RYGB (3)	8.7	46.4	−1.13%
Mendez et al. (16)	3	25	12 months	RYGB (3)	16.5	56.33	+0.07%
Fuertes-Zamorano et al. (17)	2	29.5	54 months	BPD (2)	23	1st patient: 0.23 u/k/d 2nd patient: 0.29 u/kg/d	−1.4%
Chuang et al. (18)	2	5.5	20 months	RYGB (1) SG (1)	18.1	1st patient: −25 IU/day (+0.1 u/kg/d) 2nd patient: −40 IU/day (no difference in u/kg/d)	1st patient: +0.2% 2nd patient: +3.7%
Tang et al. (19)	6	18	16 months	AGB (3) SG (2) RYGB (1)	11.4	Reduced in all cases	+0.1%
Brethauer et al. (20)	10	22	36.8 months	RYGB (7) AGB (2) SG (1)	11.1	0.34 u/kg/d	−1.1%
Lannoo et al. (21)	22	25.3	37.8 months	RYGB (16) SG (6)	8.3	44.5	−0.2% (non-significant)
Landau et al. (22)	26	20.2	3.5 years	SG (19) RYGB (4) AGB (3)	8.4	NA	+0.1%
Vilarrasa et al. (23)	32	20	4.6 years	SG (15) RYGB (11) BPD (6)	9	50.9	−0.6% at 12 months, return to baseline at endpoint
Raab et al. (24)	6	17.16	20 months	BPD (3) RYGB (2) SG (1)	13.8	67.4	−1.23%
Robert et al. (25)	10	23.1	55.1 months	BPD (7) SG (3)	16.56	0.65 u/kg/d	−0.4% (non-significant)
Blanco et al. (26)	7	27.3	24 months	RYGB (7)	12.1	+ 0.1 u/kg/d	−0.1% (non-significant)
Reyes- Garcia et al. (27)	1	24	10 months	RYGB (1)	14.6	Reduction	1.3%
Rottenstreich et al. (28)	13	19.1	24 months	SG (10) RYGB (3)	8.8	38	−0.8% (non-significant)
Middelbeek et al. (29)	10	24.6	5 years	RYGB (10)	9.7	21.9	+1.7%
Maraka et al. (30)	7	20.6	2 years	RYGB (9) SG (1)	13.1	19.7	−0.4%
Faucher et al. (31)	13	21	12 months	SG (7) RYGB (6)	11.5	0.35 u/kg/d	−0.7%
Al Sabah et al. (32)	10	NA	4 years	SG (10)	10.5	58.4	−0.3% (non-significant)
Moreno-Fernandez et al. (33)	6	24.2	4.5 years	RYGB (3) SG (3)	14.2	16.1	−0.6% (non-significant)

*RYGB, R-n-Y gastric bypass; SG, sleeve gastrectomy; BPD, biliopancreatic diversion; AGB, adjustable gastric banding; IU, international units; NA, not applicable; HbA1c, glycosylated hemoglobin A1c; T1DM, type 1 diabetes mellitus*.

### Outcomes in Weight Loss

Bariatric surgery offers a significant reduction in body mass index (BMI) in patients with obesity and T1DM which is sustained in long term. Czupryniak et al. (15) were the first to show a marked improvement in the mean BMI of three women with obesity who underwent RYGB from 42.2 kg/m^2^ preoperatively to 33.5 kg/m^2^ post-operatively in a follow-up period of ~7 years. After an ~3-years follow-up time, mean reductions in BMI were 11.1, 8.3, and 9.4 kg/m^2^ in other cohorts (20–22). In a relatively large cohort of 32 subjects with obesity studied for 4.6 years, the mean percentage of total weight loss (%TWL) at 12 months after surgery was 30.4%, with a slight decrease at 5 years, reaching 28.1% (23). Cumulatively, a meta-analysis by Chow et al. (34), including 86 patients in total, showed a reduction in BMI of 13.42 kg/m^2^ post-operatively. Another meta-analysis by Ashrafian et al. (35), including 142 patients in total, showed a mean BMI decrease of 11.04 kg/m^2^, in a mean follow-up time of 31.8 months. In general, similar results have been demonstrated by every study on the field regardless of the type of intervention applied, the number of patients or the follow-up period (16–19, 24–32, 36).

### Outcomes in Insulin Requirements

The reduction in body weight after surgery is accompanied by a reduction in total daily insulin requirements in patients with obesity and T1DM. Middelbeek et al. (29) evaluated the post-surgical outcomes in 10 women with T1DM at 1 and 5 years after RYGB. Daily insulin requirements were decreased from 53 to 23 units/day at 1 year, with a slight increase in 31.1 units/day at 5 years, presenting a strong correlation with body weight which followed a similar trend. In accordance with weight loss outcomes, insulin requirements were reduced in the reports by Brethauer et al. (20) and Lannoo et al. (21), with a mean change of 0.34 units/kg/day and 56.33 units/day, respectively. In a study by Robert et al. (25), where the procedures applied on 10 patients with T1DM were either biliopancreatic diversion (BPD) or sleeve gastrectomy (SG), the daily insulin dose was diminished from 1.09 to 0.44 units/kg/day during a mean follow-up of 55.1 months. The sustained reduction in insulin dose is confirmed in the meta-analyses mentioned above (34, 35) where, irrespectively of the follow-up period, the post-operative decrease in insulin requirements was 42 units/day and 0.391 units/kg/day, respectively.

### Outcomes in HbA1c

In contrast to the results concerning weight loss and insulin requirements, the effects of bariatric surgery on HbA1c seem to be inconsistent among studies. Czupryniak et al. (15) and Raab et al. (24) showed a statistically significant mean decrease in HbA1c of 1.13 and 1.23% at 7 and 1 year after surgery, respectively. Similar results were shown by Brethauer et al. (20), where the mean decrease was 1.1%, and by Fuertes-Zamorano et al. (17), who showed a mean decrease of 1% in two patients who underwent BPD at 5 years post-surgery. However, these favorable outcomes come into contradiction with those in other studies. In the study by Middelbeek et al. (29), the HbA1c was unchanged at 1 year after surgery (8.1–8.3%) and, even more interestingly, it was increased at 5 years post-operatively to 9.8%. This transient, modest improvement was observed in other studies (21–23, 26, 28). Overall, as the available meta-analyses indicate, the average mean change in HbA1c ranges from 0.64 to 0.93% (34, 35); in any case, the optimal target of ≤ 7% is not reached, meaning that bariatric surgery seems to be barely beneficial in terms of glycemic control. The reasons for this discrepancy are yet to be explained and are discussed below.

### Outcomes in Co-morbidities

The evidence about the impact of bariatric surgery on the common metabolic co-morbidities that accompany T1DM combined with excess weight (so called “double diabetes”) is scarce, as many of the already limited studies on the field did not include such risk factors in their endpoints. However, some existing data is encouraging (Table 2). Brethauer et al. (20) showed a reduction in the levels of low-density lipoprotein (LDL) and triglycerides (TG) of 23 and 30.5 mg/dl, respectively, along with an increase in high-density lipoprotein (HDL) of 10.8 mg/dl. Hypertension resolved in 5 out of 7 hypertensive patients and albuminuria resolved in 1 out of 2 patients who presented microalbuminuria preoperatively. Chuang et al. (18) reported on two adolescents who underwent bariatric surgery. The first one achieved a significant amelioration in his lipid profile (LDL decreased from 180 mg/dl to 81 mg/dl and HDL increased from 32 to 45 mg/dl at 12 months after surgery) and quit statin agents, while he presented with a tremendous improvement in his obstructive sleep apnea. The second patient, suffering from polycystic ovarian syndrome (PCOS) as well, presented a similar improvement in lipids (LDL decreased from 109 to 73 mg/dl and HDL increased from 41 mg/dl to 59 mg/dl at 28 months after surgery) and managed to stop oral contraceptives and restore regular menses. In a long-term cohort, the number of patients with dyslipidemia, obstructive sleep apnea, hypertension and microalbuminuria was decreased by 25, 66, 42.8, and 25%, respectively (23). A considerable decrease of 26.9 mg/dl in triglycerides was observed by Fernandez (33), who studied 6 patients who underwent either RYGB or SG for a mean follow-up of 4.5 years. In the systematic review by Ashrafian (35), it was estimated that the mean decreases in systolic blood pressure (SBP) and diastolic blood pressure (DBP) were 10.1 and 6.193 mmHg, respectively. Regarding lipid profile, LDL and TGs were reduced by a 9.54 and 11.04 mg/dl, whereas HDL was increased by a mean 13.51 mg/dl. It must be noted, however, that these results were extracted from a very limited number of studies of acceptable quality.

**Table 2:** Effects of bariatric surgery on metabolic comorbidities in patients with obesity and type 1 diabetes mellitus.

**References**	**Patients (*n*)**	**Mean duration of follow-up**	**Procedures (*n*)**	**Effects on co-morbidities**
Czupryniak et al. (15)	3	75.6 months	RYGB (3)	Improvements in blood pressure, lipid profile, microalbuminuria
Fuertes-Zamorano et al. (17)	2	54 months	BPD (2)	1st patient: resolution of dyslipidemia 2nd patient: resolution of dyslipidemia and hypertension
Chuang et al. (18)	2	20 months	RYGB (1) SG (1)	1st patient: no treatment for dyslipidemia, sleep apnea 2nd patient: restoration of regular menses, stop OCP
Brethauer et al. (20)	10	36.8 months	RYGB (7) AGB (2) SG (1)	Improvements in LDL (−23 ± 19.3 mg/dl), HDL (10.8 ± 3.4 mg/dl), TG (−30.5 ± 17.1 mg/dl). Resolution of HTN in 5/7 patients, resolution of albuminuria in 1/2 patients.
Landau et al. (22)	26	3.5 years	SG (19) RYGB (4) AGB (3)	Improvements in hypertension, HDL increase
Vilarrasa et al. (23)	32	4.6 years	SG (15) RYGB (11) BPD (6)	Remission of HTN, dyslipidemia and OSA in 42.8, 25, and 66%, respectively. Regression of microalbuminuria to normoalbuminuria in 25%.
Robert et al. (25)	10	55.1 months	BPD (7) SG (3)	Remission of hypertension in 66.7%, dyslipidemia in 88.9%
Rottenstreich et al. (28)	13	24 months	SG (10) RYGB (3)	Resolution of hypertension in 4 patients
Middelbeek et al. (29)	10	5 years	RYGB (10)	HDL increase by 22% at 5 years. Improvements in triglycerides and SBP at 1 year but not at 5 years
Maraka et al. (30)	7	2 years	RYGB (9) SG (1)	Decrease in lipid-lowering medications
Moreno-Fernandez et al. (33)	6	4.5 years	RYGB (3) SG (3)	Remission of HTN in 1/3 patients. Improvements in TG (−26.9 ± 15.1 mg/dl)

*HDL, high-density lipoprotein; OCP, oral contraceptives; SBP, systolic blood pressure; OSA, obstructive sleep apnea; HTN, hypertension; LDL, low-density lipoprotein; TG, triglycerides; RYGB, R-n-Y gastric bypass; SG, sleeve gastrectomy; BPD, biliopancreatic diversion; AGB, adjustable gastric banding*.

## Complications of Bariatric Surgery

Bariatric surgery procedures are generally considered safe. The average perioperative mortality is <0.3%, comparable to this of appendicectomy or cholecystectomy. The 1-year complication rate varies among different procedures, being 4.6, 10.8, 14.9, and 25.7% for AGB, VSG, RYGB, and BPD, respectively (37). The factors that negatively affect the incidence of adverse events after surgery include male sex, the presence of co-morbidities, high pre-operative BMI, old age, and the lack of adequate experience of the surgical team. Among patients with T1DM, no excess in mortality has been reported (38). Apart from the common complications of bariatric surgery, however, an increased prevalence of diabetic ketoacidosis (DKA) and hypoglycemia has been observed (Table 3).

**Table 3:** Incidence of diabetic ketoacidosis and hypoglycemia after bariatric surgery in patients with type 1 diabetes mellitus and obesity.

**References**	**Patients (*n*)**	**Mean duration of T1DM (years)**	**Mean duration of follow-up**	**Procedures (*n*)**	**DKA before surgery**	**DKA after surgery**	**Hypoglycemia before surgery**	**Hypoglycemia after surgery**
Chuang et al. (18)	2	5.5	20 months	RYGB (1) SG (1)	Patient 1: 1 episode Patient 2: (–)	Patient 1: (–) Patient 2: 1 episode	(–)	Patient 1: Mild hypoglycemic episodes 2 to 5 times per week Patient 2: Mild hypoglycemic episodes 2 to 5 times per week
Aminian et al. (39)	12	NA	90 days	RYGB (6) SG (4) AGB (2)	3 patients (25%)	Severe: 3 Moderate: 3 Mild: 6	NA	NA
Maraka et al. (30)	7	20.6	2 years	RYGB (9) SG (1)	NA	2 patients	NA	7 patients
Vilarrasa et al. (23)	32	20	4.6 years	SG (15) RYGB (11) BPD (6)	(-)	2 patients (one had a recurrent episode)	(-)	3 patients (9.3%)—severe
Landau et al. (22)	26	20.2	3.5 years	SG (19) RYGB (4) AGB (3)	NA	4 patients (within 48 h after surgery)	NA	6 patients shortly after discharge−2 patients hospitalized
Faucher et al. (31)	13	21	12 months	SG (7) RYGB (6)	NA	NA	(-)	Median number of minor episodes at 6 months: RYGB: 3.5 SG: 6 2 severe episodes
Al Sabah et al. (32)	10	NA	4 years	SG (10)	NA	NA	NA	2 patients (18.2%)

*DKA, diabetic ketoacidosis; NA, not applicable; RYGB, R-n-Y gastric bypass; SG, sleeve gastrectomy; BPD, biliopancreatic diversion; AGB, adjustable gastric banding*.

### Diabetic Ketoacidosis

Diabetic ketoacidosis is a life-threatening condition which is not uncommon in patients with T1DM after bariatric surgery. In a study by Aminian et al. (39), the incidence of DKA in the early post-operative period (0–61 days) in T1DM patients was reported to be as high as 25%. Three out of the eight T1DM patients who developed ketoacidosis had a previous episode and all of them had poor glycemic control preoperatively with a mean HbA1c of 9.3%. In another cohort, 15% of patients had an episode of DKA within 48 h after surgery due to insufficient insulin dosage post-operatively (22). Similar results were reported by Maraka et al. (30), where 20% of T1DM patients who underwent surgery had an episode of ketoacidosis contrary to no incidents in the T2DM group, while in the group of Rottenstreich et al. (28), the incidence of DKA within the first months after surgery was 21.4%. However, DKA poses as a threat even in the long term; 3 out of a total of 32 T1DM patients studied for almost 4.5 years presented with episodes of ketoacidosis within 3 years after surgery (23). Apart from the obviously high prevalence of this complication, another interesting fact is that, in the setting of the reduced carbohydrate intake that follows surgery along with the continued insulin regimen, diabetic ketoacidosis may not necessarily present with hyperglycemia, as in the recently reported case of a 43 years-old woman who developed euglycemic diabetic ketoacidosis 10 days after she underwent sleeve gastrectomy (40). Rather than altered glucose kinetics, it seems that poor peri-operative glycemic control with discontinuation or inadequate intake of insulin is the main precipitating factor that leads to DKA; others include anesthesia, surgical stress, infection, and fluid and calorie deprivation (39).

### Hypoglycemia

Hypoglycemia is a relatively frequent complication in T1DM patients undergoing bariatric surgery, reaching an incidence of even 70% (30). The majority of cases pertains to the early post-operative period; in three different cohorts, the rate of hypoglycemic episodes was 18% (32), 23% (22), and 29% (28) within the first few days to months after surgery. Altered glucose kinetics, with the rapid delivery of carbohydrates to the jejunum create an imbalance between the higher and earlier glucose excursions and the action of exogenous insulin, while the improvement in insulin sensitivity that follows weight loss renders the previously needed insulin dosages to become excessive. However, no surgical procedure seems to prevent such episodes, despite the fact that sleeve gastrectomy is considered, in general, to be safer due to a more predictable pattern of carbohydrate absorption. Among 32 patients who underwent either biliopancreatic diversion, sleeve gastrectomy or R-n-Y gastric bypass, three patients (9.3%) developed severe hypoglycemic episodes within the first year after surgery, one after each procedure applied (23). In a study by Faucher et al. (31), a median of four minor hypoglycemic episodes per week was reported at 6 months after surgery, with more cases being associated with sleeve gastrectomy (median 6 cases) rather than R-n-Y gastric bypass (median 3.5 cases). It is therefore evident that a close monitoring of glucose levels along with careful insulin titration according to the carbohydrate load of each meal is of vital importance in the early post-operative period in order to prevent hypoglycemic episodes regardless of the surgical method used. In this respect, sensor-augmented insulin pump therapy with automated insulin suspension has been related to more stable glycemic control in some patients (16, 26), however, adequate data regarding the most efficient type of insulin after surgery is not available.

### Other Complications

As mentioned above, the rate of surgical complication ranges from 4 to 25%, depending on the procedure applied. However, these rates are constantly declining thanks to the improvement in surgical techniques and the existence of more and more experienced bariatric surgery centers. Wound infection and post-operative bleeding, which can derive from the omental or mesenteric vessels or the anastomotic site, are the most common short-term complications after surgery (41). Pulmonary embolism is another common adverse event with a risk of 2% and, along with anastomotic leak, are the leading causes of death in the early post-operative period. Among the late complications, marginal ulcers, anastomotic stenosis, cholelithiasis, and hernias are the most frequent ones. Among the T1DM population, another common concern is the presence of autonomic neuropathy and gastroparesis, which can lead to complication, such as nausea, vomiting, and acute gastric remnant dilation (38, 42).

The most common and serious complications of bariatric surgery are nutritional deficiencies, which occur mainly in malabsorptive rather than in restrictive procedures (43–46). The main nutrients affected are iron, vitamin B12, folic acid, calcium, and fat-soluble vitamins (A, D, E, K). Iron deficiency, depending on the procedure, has a risk of 13–52% within the first 5 years after surgery, and is attributed to reduced gastric acid production. Vitamin B12 deficiency can occur in up to 70% of patients after RYGB and is due to the failure of separation of B12 from food and to inadequate secretion of the intrinsic factor (47, 48). Folic acid deficiency is less frequent (up to 35% in patients after RYGB) and its causes include achlorhydria, bypassing of the duodenum, reduced dietary intake and vitamin B12 deficiency (48, 49). As calcium is preferentially absorbed in the duodenum and proximal jejunum, the exclusion of these segments leads to malabsorption and hypocalcemia, whose incidence can reach up to 10% after surgery, while the rate of vitamin D deficiency is up to 51% (50, 51). The result is the development of secondary hyperparathyroidism, which leads to increased release of calcium from bones and, eventually, loss of bone mass and osteoporosis (52–54). Regardless of the operation performed, all patients who have undergone bariatric surgery should follow a close nutritional monitoring on a regular basis, receiving appropriate supplements when necessary.

Finally, the most recent concern regarding post-surgical complications are psychosocial disorders (55). As the gastrointestinal anatomy after RYGB and SG favors the rapid absorption of alcohol, the rate of alcohol misuse events in these patients is increased and poses as a substitute to their previous food addiction (56–58). Even more alarmingly, the risk of suicide after bariatric surgery seems to rise. In the Utah Mortality Study, a significant increase in the number of suicides, poisonings and accidental deaths after RYGB was observed, and similar were the results in the second Utah Obesity Study (59, 60). In 2010, Tindle et al. (61) reported 31 suicides in a total of 16.683 bariatric surgery operations (overall rate of 6.6/10.000), with 70% of the events occurring within the first 3 years after surgery. In a review by Peterhansel et al. (62) the suicide rate after surgery was estimated to be 4.1/10.000 person-years. A similar trend was shown in 2017 by Laggeros et al. (63), where it was also indicated that episodes of self-harm and depression were more frequent after gastric bypass in patients with a pre-existing psychiatric condition. These data underline the importance of a comprehensive evaluation of the psychiatric status of patients both prior to and after surgery by a multidisciplinary team of experienced healthcare professionals, so that timely identification and management of such conditions is achieved (64).

## Glucose Homeostasis After Bariatric Surgery and Special Concerns in T1DM

As bariatric surgery has only recently been considered as a therapeutic option for T1DM, evidence concerning the possible mechanisms through which it impacts glucose metabolism has been obtained from studies on human and animal models with T2DM. Weight loss, calorie restriction and gastrointestinal hormone modulation are some of the mechanisms which have been described to play an important role in the remission of T2DM after bariatric surgery (65). The effect of these mechanisms in T1DM has not yet been elicited due to the different pathogenesis comparing to T2DM. However, the fact that bariatric surgery results in a significant reduction of insulin requirements and an, at least modest, reduction in HbA1c in T1DM patients, implies the existence of a shared background between these two types of the disease on which surgery exerts mutually beneficial effects. The discrepancies, nevertheless, in the post-operative glycemic control in T1DM are a serious concern that needs to be elucidated. To discuss these concerns, a brief presentation of the main mechanisms through which surgery acts on glucose metabolism is required.

### Mechanisms of Glycemic Improvement

#### Beta Cell Function and Insulin Secretion

Bariatric surgery attenuates the dysfunction of beta cells by increasing beta cell sensitivity (66). As it is obvious, in the setting of T1DM, such an action is meaningful only in cases of residual beta cell function. The physiological pathways which mediate this enhancement have been poorly clarified. Caloric restriction, removal of glucose toxicity and gastrointestinal hormone modulation have been proposed; however, it seems that a main mechanism is the enhanced glucagon-like peptide-1 (GLP-1) secretion after surgery. The rapid and early delivery of nutrients to the distal small intestine potentiates the secretion of distal gut peptides, mainly glucagon-like peptide-1 (GLP-1) and peptide YY (PYY) (67). Post-prandial concentration of GLP-1 rises almost 10-fold in the first few days after RYGB, and similar increases are observed after SG and BPD, but not after AGB (68–70). The importance of exaggerated post-prandial responses of GLP-1 post-operatively has not been studied specifically in T1DM patients after metabolic surgery, however it has been confirmed in studies where the GLP-1 receptor was blocked with exendin (9–39) and blunted insulin responses were reported after meals (71, 72). In a study by Scrocchi et al. (73), a defective glucose-stimulated insulin secretion was noted in mice which were deprived of the GLP-1 receptor in β-cells (GLP-1R^−/−^), while in another murine model of diabetes, the administration of GLP-1R agonist exendin-4 attenuated translational downregulation of insulin and improved beta cell survival (74). Similarly, in a tamoxifen-inducible GLP-1R knockout mouse model, augmentation of glucose-stimulated insulin secretion during an oral glucose tolerance test (OGTT) after VSG was blunted in the knockout mice compared to the control group (75); such results were reproduced in another study on humans after RYGB, where administration of exendin 9–39 decreased insulin concentrations (12.3 ± 2.2 vs. 18.1 ± 3.1 nmol/6 h) and β-cell response to glucose post-prandially compared to controls (76). In addition, some researchers have attributed post-RYGB hyperinsulinemic hypoglycemia to GLP-1-mediated expansion of β-cell mass (nesidioblastosis) (77, 78), with reversal of the symptoms after blockade of GLP-1 receptor with exendin 9–39 (79). Contrary to these results, a human study comparing RYGB patients with a group undergoing an intensive lifestyle modification therapy showed no differences in glucose tolerance deterioration after the infusion of exendin 9–39 (80). Similar results were demonstrated in animal studies, where the infusion of exendin 9–39 in GLP-1R^−/−^ and wild type mice which underwent RYGB or VSG resulted in similar glucose tolerance (81, 82). Therefore, the role of enhanced GLP-1 secretion after bariatric surgery on β-cell function remains to be clarified. As for the other major incretin, glucose-dependent insulinotropic polypeptide (GIP), evidence is inconsistent, with studies showing contradictory responses after either SG or RYGB (83–85).

#### Hepatic and Peripheral Insulin Resistance

The improvement of insulin sensitivity in the early post-operative period is attributed to enhanced hepatic insulin sensitivity (86). This is achieved via a reduction in the liver fat content through increased lipolysis and lipid oxidation (87). It seems that caloric restriction, which occurs immediately after all types of surgical procedures, is the main factor that leads to enhanced hepatic insulin sensitivity. In a study where patients who underwent RYGB were compared with a group who consumed the same very low-calorie diet (VLCD) of 200–300 kcal/day, the improvement in HOMA insulin resistance was 25% 4 days after surgery and similar between the two groups (88). Similar were the results in two other studies, where changes in insulin sensitivity and insulin secretion were either similar or even superior in the first days after RYGB compared to VLCD (89, 90). In another study by Gastaldelli et al. (91), insulin sensitivity was estimated in a group of 20 patients using the more precise method of the hyperinsulinemic-euglycemic clamp, conducted 1 week after a very-low-calorie diet and 1 week after RYGB or LAGB. Both types of surgery led to substantial improvements in hepatic insulin sensitivity and lipolysis compared to diet alone, while RYGB also led to enhanced adipose insulin sensitivity and M/I (peripheral insulin sensitivity divided by the steady-state plasma insulin concentration). Similar results were demonstrated in another study with human subjects, where the hyperinsulinemic-euglycemic clamp 1 week after RYGB showed enhanced hepatic insulin sensitivity and increased insulin clearance (86). On the other hand, in the late post-operative period, peripheral insulin sensitivity is augmented as a result of the substantial weight loss that has been achieved at that point, and a positive correlation has been observed (68, 87). Muscelli et al. (92), using the insulin clamp technique in patients who underwent either RYGB or BPD, showed an enhancement in peripheral insulin sensitivity both in the RYGB group (23.8 ± 6.0 μmol/min/kg at 5 months and 33.7 ± 11.3 μmol/min/kg at 16 months) and in the BPD group (52.5 ± 12.4 μmol/min/kg at 6 months and 68.7 ± 9.5 μmol/min/kg at 24 months) after comparable weight loss (53 kg on average). In agreement with these findings, Campos et al. (93) compared patients who underwent RYGB to patients who remained on diet alone at 14 days and 6 months post-surgery, and showed that peripheral glucose uptake, despite being similar at 14 days, was substantially enhanced only in the RYGB group at 6 months. This enhancement is, again, multifactorial. The increase in anorexigenic hormones (GLP-1, PYY, oxyntomodulin) induces early satiety and limited calorie intake. GLP-1 affects the brain indirectly, through vagal nerve fibers in the intestine, whereby it transmits various signals to the nucleus of the solitary tract (NTS), an area in the brain which plays a crucial role in regulating feeding patterns (94). GLP-1R signaling attenuates the release of the orexigenic neuropeptides Neuropeptide Y (NPY) and agouti-related peptide (AgRP), and enhances the release of the anorexigenic neuropeptides pro-opiomelanocortin (POMC) and cocaine- and amphetamine-regulated transcript (CART) (95, 96); it increases thermogenesis in brown adipose tissue (BAT) through enhancing sympathetic nervous system activity (97); and inhibits neuronal activity in reward processing areas as functional magnetic resonance imaging (fMRI) studies in obese and T2DM patients have shown (98). PYY levels are parallel to those of GLP-1, and therefore, are also increased after gastric bypass but not after gastric banding (99, 100). In addition, as studies in both human and animal models have shown, the levels of bile acids are increased after RYGB (but not after SG or AGB), leading to increased secretion of GLP-1 through the TGR5 receptor on L-cells, while they also induce synthesis of fibroblast growth factor 19 (FGF19) on ileal enterocytes which enhances insulin sensitivity through boosting mitochondrial activity (101–104). Finally, metabolic surgery does not contribute only to the decrease of BMI in general, but actually leads to a redistribution of overall body fat mass, while the visceral and intramuscular depot are also reduced, effects which are also mediated, at least in part, by GLP-1, which can induce increased thermogenesis in BAT through various molecular pathways (65, 94, 105). The improvement in visceral fat and glucose uptake by the skeletal muscle is a result of enhanced insulin sensitivity and the increased GLP-1 levels, which promote glycogen synthesis, glycogen synthase α (GSα) activity and glucose metabolism and, on the other hand, inhibit glycogen phosphorylase α activity, as various studies in both animal and human models have demonstrated (94), while these morphological changes in adipose tissue lead to decreased levels of leptin and other pro-inflammatory cytokines and increased levels of adiponectin (106, 107). The role of gut microbiota in insulin sensitivity is still to be elucidated (101, 108).

#### Glucose Metabolism

Glucose absorption is diminished after RYGB and VSG. Regarding RYGB, altered bile acids kinetics lead to the absence of sodium in the alimentary limb, which is necessary for the endoluminal absorption of glucose via the apical sodium glucose cotransporter 1 (SGLT1) (109), while regarding SG, the reduction in glucose absorption is attributed to the reduced leptin- and ghrelin-expressing cells (65). Ghrelin is an orexigenic gut hormone that is produced in gastric fundus and whose post-prandial suppression is diminished in individuals with obesity. SG decreases both fasting and post-prandial levels of ghrelin, but such suppression has been shown in RYGB as well, while other studies have shown no change or even an increase (110–112). In addition, after RYGB (but not after SG), the alimentary limb presents mucosal hypertrophy and hyperplasia, which amplifies glucose utilization from the gut (113). Finally, it has been postulated that the exclusion of nutrients from the duodenum eliminates an, unknown, “factor” with anti-incretin properties which leads to hyperglycemia (101). In a study in mice, the group which underwent RYGB presented an increased activity of phosphoenolpyruvate carboxykinase (PEPCK) and glucose-6-phosphatase (Glc6Pase) enzymes in the distal jejunum and ileum, thus promoting intestinal gluconeogenesis raising glucose concentration in the portal vein, an effect which is potentiated by GLP-1 and eventually leads to suppressed hepatic glucose production and improved glucose tolerance. These effects were not observed in mice treated with gastric banding (which does not cause redirection of nutrients), as well as in mice with downregulation of the glucose transporter 2 (GLUT2), which is essential for hepato-portal sensing and is stimulated after RYGB (114, 115).

### Glycemic Control in T1DM: Who Benefits?

As the above discussed mechanisms reveal, the major pathway through which bariatric surgery beneficially affects glycemic control in T1DM is the enhancement in insulin sensitivity. “Double diabetes” (DD) is the term used to describe the type of the disease which is characterized by the occurrence of factors typical of both type 1 and type 2 diabetes; insulin resistance and autoimmunity against beta cells (116). DD can occur when a patient with T2DM develops autoantibodies against beta cells or when an individual with T1DM becomes obese and consequently develops hepatic and peripheral insulin resistance. Nevertheless, glycemic control after bariatric surgery is far from optimal, and the explanation for this is probably multifactorial. Firstly, in some studies, post-operative inhibition of glucagon secretion was not detected despite the increased GLP-1 secretion. In the study by Blanco et al. (26), where seven women with T1DM were compared with seven women with T2DM for 24 months after RYGB, the similarly enlarged post-prandial GLP-1 secretion between the two groups was not accompanied by suppression but rather by an increase in glucagon secretion. Similar were the findings by Dirksen et al. (117) in a patient at 12 months after RYGB, where the 7-fold increase in GLP-1 was accompanied by an only slight decrease in post-prandial glucagon levels. In addition, the absence of residual β-cell function in the majority of T1DM patients renders any mechanism that improves insulin secretion useless. The three available studies which directly compared the effects of surgery in T1DM and T2DM patients demonstrated impressive decreases in HbA1c in patients with C-peptide positive T2DM requiring insulin therapy but not in those with C-peptide negative T1DM (25, 26, 30). However, as a number of investigators have reported the presence of residual insulin-positive beta cells in patients with T1DM (118, 119), timing of surgery becomes crucial, as GLP-1-mediated effects can help preserve beta cell mass and delay progression to total insulin deficiency if the operation is conducted early in the course of the disease.

Another cause of “remission” of diabetes and exacerbations in glycemic control after an initial improvement in HbA1c after surgery is that the type of diabetes is often misdiagnosed, with many patients actually suffering from latent autoimmune diabetes of the adult (LADA). LADA is defined as a type of T1DM which is characterized by slow development and partial autoimmune destruction of beta cell with onset between 30 and 55 years of age (120). LADA accounts for 9–25% of adults with diabetes in the general population; in a large meta-analysis on bariatric surgery outcomes on T1DM, the rate of patients with LADA was 3.3%. The diagnosis of LADA is based on low fasting and meal-stimulated C-peptide levels and on existence of antibodies against glutamic acid decarboxylase (GAD65), insulin and/or islet cells. The secretory burden on beta cells elicited by insulin resistance leads to their subsequent apoptosis if the burden is prolonged and this is why at the early stages, adequate glycemic control can be achieved with antidiabetic oral medication, whereas in the progression of the disease insulin will be eventually required (121). Robert et al. (25) have reported a case with latent autoimmune diabetes who weaned off insulin 2 years after bariatric surgery. In the literature a case has also been reported in which sleeve gastrectomy induced remission of slowly progressive type 1 diabetes in an patient with obesity (122), with the most impressive results observed in a Japanese cohort of 5 patients with a low mean titer of anti-glutamic acid decarboxylase antibody, where mean HbA1c was reduced from 8.4 to 5.7% 1 year after sleeve gastrectomy with or without duodenojejunal bypass (123). This evidence highlights the importance of meticulous screening for LADA pre-operatively, especially in patients with poorly controlled diabetes on oral antidiabetic medication or with a history of DKA or autoimmune diseases, as early surgical intervention to preserve residual beta cell mass and function is even more important in these patients, who are the best candidates for metabolic surgery among T1DM patients. The surgery of choice is still unclear, however, even in these patients; while Robert et al. (25) showed that BPD was superior to SG in terms of weight loss, insulin requirements and resolution of dyslipidemia, other studies (124) suggest that RYGB offer the best risk-to-benefit ratio.

## Conclusion

The prevalence of obesity and metabolic syndrome in patients with T1DM has increased significantly. The term “Double Diabetes” has been introduced to describe this type of diabetes in which factors typical of both type 1 and type 2 diabetes co-exist. Bariatric surgery has been suggested as an effective treatment for these patients, although data is scarce, based on case series and retrospective studies. Bariatric surgery results in a significant reduction in body mass index, in total daily insulin requirements and in co-morbidities related to obesity. However, its effect on glycemic control in T1DM is controversial, as sustainable and meaningful reductions in HbA1c have not been invariably achieved. Apart from the common complications of BS, diabetic ketoacidosis and hypoglycemia are life threatening conditions with increased prevalence in T1DM patients, which require special concern and whose causal mechanisms are still unclear. Improvement in beta cell function, reduction of hepatic and peripheral resistance and modifications in glucose absorption are some of the mechanisms through which BS improves glycemic control in both type 1 and type 2 diabetes suggesting that the two types of diabetes have partially a common pathophysiological pathway; however, the inadequate glycemic control post-surgery has not yet been explained. In conclusion, bariatric surgery is not yet to be recommended routinely to all T1DM patients. For the time being, those patients with LADA considered the most appropriate candidates for surgery when the operation is conducted at the early stages of the disease, as the surgical intervention preserves beta cell mass and delays progression to total insulin deficiency. For all the other T1DM patients, a careful and multidisciplinary approach is necessary, where individualized and realistic goals will be set. Randomized, prospective trials are required to conclusively evaluate the effects of surgery in T1DM both in short and long term and define the most suitable candidates who will benefit the most out of it.

## Author Contributions

All authors listed have made a substantial, direct and intellectual contribution to the work, and approved it for publication.

## Conflict of Interest

The authors declare that the research was conducted in the absence of any commercial or financial relationships that could be construed as a potential conflict of interest.

## Glossary

AGB: adjustable gastric banding
AgRP: agouti-related peptide
BAT: brown adipose tissue
BMI: body mass index
BPD: biliopancreatic diversion
DBP: diastolic blood pressure
CART: cocaine- and amphetamine-regulated transcript
DD: double diabetes
DKA: diabetic ketoacidosis
FGF19: fibroblast growth factor 19
fMRI: functional magnetic resonance imaging
GAD65: glutamic acid decarboxylase
Glc6Pase: glucose-6-phosphatase
GLP-1: glucagon-like peptide-1
GLP-1R: glucagon-like peptide-1 receptor
GLUT2: glucose transporter 2
HbA1c: glycosylated hemoglobin A1c
HDL: high-density lipoprotein
IGT: impaired glucose tolerance
IUs: international units
LADA: latent autoimmune diabetes of the adult
LDL: low-density lipoprotein
NPY: Neuropeptide Y
NTS: nucleus of the solitary tract
OGTT: oral glucose tolerance test
PCOS: polycystic ovarian syndrome
PEPCK: phosphoenolpyruvate carboxykinase
POMC: pro-opiomelanocortin
PYY: peptide YY
RYGB: Roux-en-Y gastric bypass
SBP: systolic blood pressure
SGLT1: glucose cotransporter 1
T1DM: type 1 diabetes mellitus
T2DM: type 2 diabetes mellitus
TGs: triglycerides
TWL: total weight loss
VLCD: very low-calorie diet
VSG: vertical sleeve gastrectomy
